# Preprocedural mouthwashes for infection control in dentistry—an update

**DOI:** 10.1007/s00784-023-04953-z

**Published:** 2023-04-20

**Authors:** Johanna Weber, Eva L. Bonn, David L. Auer, Christian Kirschneck, Wolfgang Buchalla, Konstantin J. Scholz, Fabian Cieplik

**Affiliations:** 1 Department of Conservative Dentistry and Periodontology, University Hospital Regensburg, Franz-Josef-Strauß-Allee 11, 93053 Regensburg, Germany; 2 Department of Orthodontics, University Hospital Regensburg, Regensburg, Germany

**Keywords:** Mouthwash, Mouth rinse, Infection control, SARS-CoV-2

## Abstract

**Objectives:**

Aerosols and splatter are routinely generated in dental practice and can be contaminated by potentially harmful bacteria or viruses such as SARS-CoV-2. Therefore, preprocedural mouthwashes containing antiseptic agents have been proposed as a potential measure for infection control in dental practice. This review article aims to summarize the clinical (and, if insufficient, preclinical) evidence on preprocedural mouthwashes containing antiseptic agents and to draw conclusions for dental practitioners.

**Methods:**

Literature on preprocedural mouthwashes for reduction of bacterial or viral load in dental aerosols was searched and summarized.

**Results:**

Preprocedural mouthwashes, particularly those containing chlorhexidine digluconate (CHX), cetylpyridinium chloride (CPC), or essential oils (EO), can significantly reduce the bacterial load in dental aerosols. With respect to viruses such as HSV-1, there are too little clinical data to draw any clear recommendations. On the other hand, clinical data is consolidating that CPC-containing mouthwashes can temporarily reduce the intraoral viral load and infectivity in SARS-CoV-2 positive individuals. Nevertheless, potential risks and side effects due to regular antiseptic use such as ecological effects or adaptation of bacteria need to be considered.

**Conclusions:**

The use of preprocedural mouthwashes containing antiseptics can be recommended according to currently available data, but further studies are needed, particularly on the effects on other viruses besides SARS-CoV-2. When selecting a specific antiseptic, the biggest data basis currently exists for CHX, CPC, EO, or combinations thereof.

**Clinical relevance:**

Preprocedural mouthwashes containing antiseptics can serve as part of a bundle of measures for protection of dental personnel despite some remaining ambiguities and in view of potential risks and side effects.

## Introduction

In contemporary dental practice, aerosols and splatter are routinely generated during various treatments by use of water-cooled rotating or oscillating instruments such as high-speed hand pieces or sonic and ultrasonic scalers [1–4]. These aerosols can contain bacteria and viruses, either originating from the patient (e.g., produced by coughing or aerosolized saliva) or from contaminated dental unit waterlines [1, 2, 4–8]. However, the actual risks resulting for health care professionals (HCPs) in dental practice due to airborne transmission of infectious diseases are still not well known [1, 2, 4, 6].

**Fig. 1:**
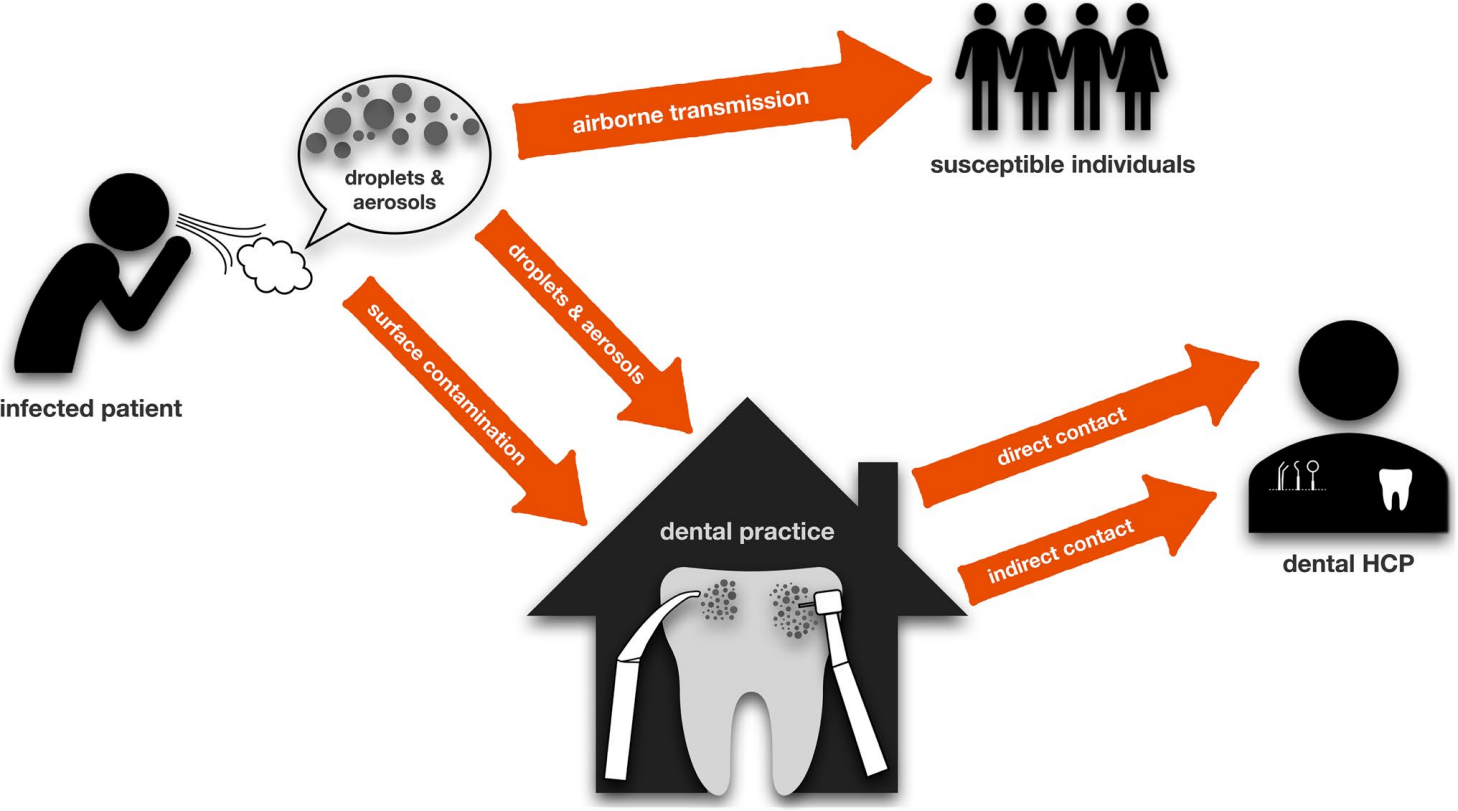
Potential transmission routes in dental practice. Most viral or bacterial diseases can be transmitted airborne by droplets or aerosols from infected patients to susceptible individuals. In dental practice, dental HCPs can get infected directly by patients, increased by the generation of aerosols during dental treatments. Furthermore, they can get infected from contaminated surfaces. This scheme has been adopted and modified from Peng et al. [18]

Rautemaa et al. investigated the spread of airborne bacteria during various dental treatment procedures [9]. They collected fall-out samples on agar plates in dental treatment rooms, where either restorative dental treatments with high-speed water-cooled rotating instruments were performed or periodontal and orthodontic treatments without use of rotating or oscillating instruments. Furthermore, face masks of HCPs and surfaces in the rooms were sampled. The results showed bacterial contamination on the face masks and at all sampling points in the rooms, which was irrespective of whether water-cooled rotating or oscillating instruments were used or not [9]. In a similar study, Zemouri et al. reported a high level of contamination centered around the patient’s head with taxa from both human and water origin [5]. Accordingly, it is well known that dental HCPs are at occupational risk of infection with *Legionella* spp. [10, 11], which are ubiquitously found in water environments [12]. Figure 1 illustrates potential transmission routes in dental practice.

Soon after introduction of antiseptics into dental practice in the 1960s [13, 14], preprocedural mouthwashes were discussed as a measure to reduce bacterial contamination of aerosols [15–17]. For instance, Mohammed et al. concluded as early as in 1964 that “an oral rinse before operative dental procedures with a high-speed drill lowers the number of microorganisms in the aerosol” [16]. About 60 years later, preprocedural mouthwashes became topical again with the outbreak of the COVID-19 pandemic caused by emergence and spread of the severe acute respiratory syndrome coronavirus 2 (SARS-CoV-2). In March 2020, Peng et al. published a review article about potential transmission routes of SARS-CoV-2 in dental practice, recommending preprocedural mouthwashes as a potential measure to temporarily reduce infectivity in SARS-CoV-2 positive patients and help protecting dental HCPs [18]. Despite sparse evidence back then, this recommendation was immediately disseminated by various other publications and professional societies [19–23], and intensively stimulated in vitro as well as clinical research activities in this area [24, 25].

The aim of this review article is to summarize the clinical (and, if insufficient, preclinical) evidence for the use of preprocedural mouthwashes with different antiseptic agents as part of a bundle of infection control measures in dentistry and to draw conclusions for dental practice.

## Antiseptics commonly used in dentistry

### Chlorhexidine digluconate

**Fig. 2:**
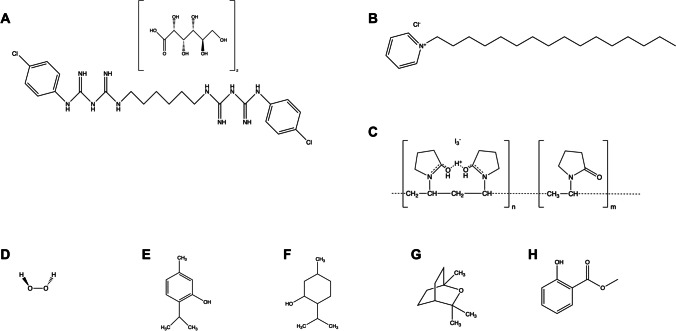
Chemical structures of antiseptics commonly used in dentistry and oral care. **A** Chlorhexidine digluconate (CHX). **B** Cetylpyridinium chloride (CPC). **C** Polyvinyl-pyrrolidone iodine (PVP-I). **D** Hydrogen peroxide (H_2_O_2_). **E** Thymol. **F** Menthol. **G** Eucalyptol. **H** Methylsalicylate

The bisbiguanide chlorhexidine (CHX; Fig. 2A) was first described in 1954 by Davies et al. as “Hibitane®” [26]. It carries two positive charges, acts as strong base, and reacts with acids forming salts, whereby today mostly the digluconate salt is used due to its superior water solubility characteristics [27, 28]. Soon after its introduction into dental practice in the 1970s, it has become the gold standard antiseptic [27, 29]. Its mechanism of action is based on damage of bacterial cytoplasmic membranes by forming hydrophilic domains followed by impairment of cellular functions and leakage of intracellular components [27, 30]. For more details, the reader may be kindly referred to a recent review article by our group [27].

### Cetylpyridinium chloride

The single positively charged quaternary ammonium compound (QAC) cetylpyridinium chloride (CPC; Fig. 2B) was first described in 1939 [31, 32]. The antimicrobial efficacy of QACs like CPC or its structural analogue benzalkonium chloride (BAC) is based on interaction with negatively charged membranes. The hydrophobic alkyl chain interacts with membranes by forming hydrophilic domains, eventually leading to leakage of cell constituents [30, 32]. Consequently, the efficacy of QACs is closely related to the hydrophobicity of the alkyl chain with peaks between C_12_ and C_16_ for both, Gram-positive and Gram-negative bacteria [33]. For more details, the reader may be kindly referred to a recent review article by our group [32].

### Povidone iodine (PVP iodine)

The use of iodine in medicine dates back to Jean Lugol’s “Lugol’s solution” in 1827 [34]. Treatment of skin or mucous membranes with aqueous or alcoholic solutions of iodine alone however was associated with irritations and excessive staining [35]. The 1952 introduced combination of the water-soluble polymer polyvinyl-pyrrolidone (PVP) with various halogens such as iodine (PVP-I; Fig. 2C) led to reduced irritational properties and toxicity accompanied by an even increased efficacy compared to the halogen use alone [35, 36]. While PVP has no antimicrobial activity, it just delivers the iodine to target cell membranes. Then, iodine is released and acts by oxidation of proteins, nucleic, or fatty acids in biological structures, resulting in membrane disruption or inhibition of metabolic pathways [34, 35, 37].

### Hydrogen peroxide

The peroxidant hydrogen peroxide (H_2_O_2_; Fig. 2D) has been used in dental practice and oral hygiene since at least 1913 [38]. It acts by oxidizing vital cell components such as lipids, proteins, and nucleic acids [35], whereby its efficacy is enhanced by the presence of metal cations such as iron or copper, which accelerate decomposition of H_2_O_2_ to hydroxyl radicals (HO•) by Fenton-like reactions [39]. H_2_O_2_ is used in various concentrations ranging from 3% up to 90%, whereby the wide distribution of catalase genes in microorganisms can increase their tolerance at low concentrations [35].

### Essential oils and herbal extracts

Mouthwashes containing herbal extracts or essential oils have been used from the nineteenth century [40]. The product mostly used in clinics (Listerine®, Johnson & Johnson, New Brunswick, NJ, USA; abbreviated as EO in this review) contains thymol (Fig. 2E), menthol (Fig. 2F), eucalyptol (Fig. 2G), and methylsalicylate (Fig. 2H) in a hydro-alcoholic solution [41, 42]. Traditionally, the mechanism of action of essential oils is thought to be based on disruption of cytoplasmic membranes and inhibition of bacterial enzymes [43–45]. However, despite many studies describing the antimicrobial activities of essential oils, herbal extracts, or their active components, there is a lack of information on their mechanisms of action [42, 45, 46]. Likewise, systematic toxicological studies are missing for EO [47].

## Preprocedural mouthwashes for infection control: bacteria

Several studies have investigated the efficacy of preprocedural mouthwashes for temporarily reducing the bacterial load in the oral cavity and in dental aerosols, as summarized in four recent systematic reviews, which, however, are partially based on different studies despite their close publication dates [48–51]. Marui et al. included 13 randomized clinical trials (RCTs) in their systematic review in which different mouthwashes containing CHX, CPC, EO, or other herbal products (made from different natural extracts such as *Mentha* spp.) were tested for their antibacterial efficacy as compared to placebo (no-rinse or rinsing with water). Twelve out of the 13 studies could prove that preprocedural mouthwashes containing these substances significantly reduced the number of bacteria in dental aerosols, resulting in a mean reduction in the number of colony forming units (CFU) by 64.8% [48]. Besides that, the meta-analysis revealed that there was hardly any difference in efficacy between CHX and CPC [48]. Only the study from Dawson et al., which focused on the effects of a preprocedural mouthwash on bacterial load and diversity in aerosols during the removal of orthodontic appliances, showed that water and CHX reduced bacterial load to the same extent [52]. Conversely, they even found higher CFU counts when rinsing with CHX than with water, which was discussed by the authors to be due to a potential dissolution of plaque caused by CHX, which in turn may increase the numbers of aerosolized bacteria [52].

In their systematic review and network meta-analysis, Koletsi et al. investigated the efficacy of preprocedural mouthwashes in reducing bacterial load during dental procedures such as ultrasonic scaling [49]. They included 21 RCTs and eight non-randomized clinical trials, whereby 11 RCTs contributed to the network meta-analysis, which compared ten different interventions. Based on the network meta-analysis, tempered CHX 0.2% at 47 °C was the most effective intervention in reducing the bacterial load measured after dental treatments with a mean CFU reduction of 92%, followed by CHX 0.2% with a mean reduction of CFU by 74% [49]. For instance, Reddy et al. demonstrated in their RCT with 30 patients that rinsing with tempered CHX had the highest efficacy as compared to non-tempered CHX or water, with CFU reduction rates of 90%, 83%, or 19%, respectively [53]. Similar results have been reported by König et al., who also concluded that tempered CHX to a temperature of 47 °C does not damage the pulp or other oral structures and thus can be used without hesitation as preprocedural mouthwash [54]. However, the individual tempering of the CHX before the respective clinical application by means of a water bath may complicate the application in the dental practice [49]. While preprocedural mouthwashes with CPC exhibited mean CFU reductions of 64% and thus were not much less effective as compared to CHX, herbal mouthwashes (including EO, tea tree oil, aloe vera extract) showed CFU reductions of 47% only [49]. Fine et al. described a 92% reduction in viable bacteria in dental aerosols for a preprocedural mouthwash with EO compared to the control when samples were taken immediately after and 40 min after rinsing [55]. Shetty et al. included 60 patients in their RCT and randomly assigned them to rinsing with distilled water, CHX, or tea tree oil before examining their efficacy in reducing the bacterial load in dental aerosols produced after a 10-min ultrasound scaling and found a significantly higher efficacy for CHX (20% reduction of CFU) than for tea tree oil (7% reduction) [56]. A recent study by Paul et al. investigated the efficacy of a preprocedural mouthwash containing 94.5% aloe vera extract as compared to 0.2% CHX and 1% PVP-I and found similar efficacy of aloe vera and CHX, which both were significantly more effective than PVP-I [57].

The third systematic review by Mohd-Said et al. examined 21 RCTs focusing on preprocedural rinsing [50]. Different mouthwashes were compared, with 18 out of 21 RCTs looking more closely at the efficacy of CHX as either a test substance or as a positive control. In seven out of 15 studies, it was also demonstrated that CHX leads to a more than 70% reduction in dental aerosols (as measured in CFU) over other tested agents such as PVP-I (two studies), CPC (three studies), and EO (two studies). In four studies, other interventions were used to examine the impact on bacterial reduction. One was the use of high-volume evacuation (HVE), the other irrigation using ozone (one study each). It was found that the HVE had an additional positive effect on reduction of bacteria load in dental aerosols generated during dental procedures [50]. Logothetis et al., for example, compared CHX and EO mouthwashes in their in vivo study. Patients were asked to perform a preprocedural mouthwash with either CHX, EO, or placebo and agar plates were placed at different locations around a reference point equivalent to the patient’s mouth. After using an air polishing device for 3 min, bacterial contamination was determined. In the group who prerinsed with CHX, the mean numbers of CFU were significantly lower at all eight locations as compared to the EO or placebo mouthwash [58].

Finally, Nagraj et al. included 17 RCTs with 830 participants in their systematic review [51]. Their primary outcome measure was incidence of infection in dental HCPs but could not be assessed because the included studies evaluated reductions of CFU as described above. They found that there was low- to very low-certainty evidence that mouthwashes containing CHX, CPC, or EO could reduce bacterial contamination. Furthermore, they also reported that there was very low-certainty evidence that tempered mouthwashes could provide a greater efficacy than non-tempered ones [51].

In summary, it can be concluded that preprocedural mouthwashes can significantly reduce the bacterial load in the oral cavity and in dental aerosols [48–51, 59]. Based on the existing data, CHX and CPC seem to be the most effective agents to be used for preprocedural mouthwashes [48–51, 59]. However, it remains unclear what size of CFU reduction represents a clinically significant amount, as also discussed by Nagraj et al. [51]. Therefore, there must be critical discussion whether CFU reductions by less than one log_10_ step can be considered relevant. Due to the exponential way of bacterial growth, antibacterial approaches usually aim for reductions by at least 3 log_10_ steps of CFU [60–62]. On the other hand, the concentrations of bacteria in aerosols are rather low and in the case of preprocedural mouthwashes just temporary effects are required, wherefore even those smaller CFU reductions in the range of 50–80% can be a good result and contribute to protection of dental HCPs.

## Preprocedural mouthwashes for infection control: viruses

In contrast to studies on the efficacy of preprocedural mouthwashes for reducing the bacterial loads in dental aerosols, there are much less studies on their effects in reducing viral loads [63–65]. Since most antiseptics used for preprocedural mouthwashes are membrane disrupting agents as described above, generally a higher efficacy is expected for inactivation of enveloped as compared to non-enveloped viruses [66]. As there is little coherent information on this topic so far, Fernandez et al. investigated in their systematic review the virucidal efficacy of CHX compared to other substances such as EO, QACs like CPC, PVP-I, or H_2_O_2_ used as mouthwash in the oral cavity [63]. While this review had some focus on SARS-CoV-2, it also included studies on other viruses such as herpes simplex virus type-1 (HSV-1; ten studies), influenza A virus (IAV; four studies), and human coronavirus (HCoV; four studies) and most of these studies agreed that CHX had virucidal effects on HSV-1 and IAV, whereas only moderate to no efficacy was found against HCoV [63].

In the in vitro study by Bernstein et al., the antiviral efficacy of 0.12% CHX mouthwash was investigated against HSV-1, cytomegalovirus (CMV), IAV, human parainfluenza virus (HPIV), poliovirus (PV), and hepatitis B virus (HBV) [67]. The 0.12% CHX mouthwash showed virucidal activity against all these viruses except PV. For instance, they found a 98% reduction in virus titer against IAV and a 99.9% reduction against HSV-1 after exposure periods of 15 min, with efficacy increasing with time. Applying the same CHX mouthwash for 30 s, the percentage of reduction was 97% only with respect to HSV-1. The inefficacy toward PV may be due to the fact that it is a non-enveloped virus [67]. On the other hand, Kanawa et al. found that PV could be inactivated by PVP-I in vitro, concluding that PVP-I may have a wider virucidal spectrum, covering both enveloped and non-enveloped viruses [68].

Baqui et al. compared in their in vitro study the antiviral efficacy of four mouthwashes, two containing EO and two containing CHX (0.12% or 0.2%), on human immunodeficiency virus type-1 (HIV-1) and HSV-1 [64]. Strains of both viruses were treated with the antiseptics for 30 s and antiviral efficacy was assessed by inhibition of syncytia formation and detection of cytopathic effects for HIV-1 on MT-2 cells and by inhibition of plaque formation for HSV-1 on Vero cells. The results showed that all tested mouthwashes inhibited both HSV-1 and HIV-1, when used undiluted or up to dilution factors of 1:2 for EO or 1:4 for CHX mouthwashes. Consequently, the authors concluded that clinical trials confirming these in vitro data would support the use of preprocedural mouthwashes for reducing viral contamination of aerosols during delivering dental care [64].

In one of the very few RCTs, Meiller et al. investigated the efficacy of a EO mouthwash in reducing infectious viral levels in saliva during an active herpes labialis infection caused by HSV-1 at stages 1 and 2, when viral shedding is highest [65]. Eighty patients were included, divided in two trials of 40 patients each. All patients gave a baseline saliva sample and were asked to rinse with the EO mouthwash or sterile distilled water as negative control for 30 s. Then, saliva samples were collected immediately after rinsing and after 30 min (trial 1) or additionally also after 60 min (trial 2). In both trials, recoverable virions were significantly reduced by about 5 log_10_ steps after the EO mouthwash with 18 out of 20 patients in each trial representing no detectable virions in the saliva samples, whereas there were no significant reductions in the control group. The EO group also demonstrated a continued significant reduction by about 3 log_10_ steps after 30 min in both trials, while in trial 2 at 60 min following the EO rinse there still was a 1–2 log_10_ step reduction as compared to baseline, which was however not significant [65].

Summarizing, there currently are too few clinical data to formulate any clear recommendations. However, there is some evidence that mouthwashes containing CHX, CPC, or EO can decrease the viral load, particularly of enveloped viruses like HSV-1, IAV, or HCoV [63, 66, 67].

## Preprocedural mouthwashes for infection control: SARS-CoV-2

Since the early stages of the COVID-19 pandemic, the use of preprocedural mouthwashes containing various antiseptics has been discussed and recommended for potentially reducing the intraoral viral load of SARS-CoV-2 during dental treatments [18, 24, 69–71].

Soon after these recommendations, several in vitro studies came out investigating a wide variety of antiseptics by exposing viral stocks of SARS-CoV-2 with the respective antiseptics to be tested for given treatment periods followed by infection of cell cultures and assessment of plaque-forming units (PFU) or 50% tissue culture infectious doses (TCID_50_) after several days of in vitro culture [24, 72–76]. These studies mostly found high virucidal efficacy for QACs like CPC or BAC, PVP-I, and EO against SARS-CoV-2, whereas CHX and H_2_O_2_ showed low efficacy [24, 72–75]. As SARS-CoV-2 is an enveloped virus, it was soon postulated that these antiseptics as membrane disrupting agents would target the viral envelope [66]. For providing experimental evidence on this hypothesis, Muñoz-Basagoiti et al. modified a commercially available ELISA quantifying the SARS-CoV-2 nucleocapsid protein [77]. This protein is located inside the viral envelope and thus can only be detected following disruption of the viral envelope. Therefore, they omitted the lysis step so that increased detection of the nucleocapsid protein indicates disruption of the viral envelope by a given active compound [77]. They found that CPC-containing mouthwashes decreased infectivity of SARS-CoV-2 and led to increased detection of nucleocapsid protein, concluding that CPC acts by disrupting the viral envelope and thus inhibiting the viral fusion with target cells [77]. Accordingly, in a study by our group, data obtained by density gradient ultracentrifugation, reverse transcription quantitative polymerase chain reaction (RT-qPCR), and nucleocapsid protection assay revealed that CPC, BAC, and PVP-I exerted their antiviral activity in vitro against SARS-CoV-2 by disruption of the viral envelope, while not affecting viral RNA [75].

Despite these promising in vitro results, the translation into clinics remains unclear, as clinical assessment of reductions in SARS-CoV-2 infectivity following use of an antiseptic mouthwash is very challenging [25]. Most currently available RCTs on this topic assessed the effects of preprocedural mouthwashes containing various antiseptics by means of RT-qPCR only [78–82], although RT-qPCR just detects viral RNA particles without giving any indication on the infectivity of these detected particles [25, 69, 75, 79, 83, 84]. Furthermore, it is known from the in vitro data that most antiseptics do not even target RNA but the viral envelope, as outlined above [75, 77, 79, 85]. Consequently, most RCTs reported no relevant reductions below 1 log_10_ step in intraoral viral loads following an antiseptic mouthwash as measured by RT-qPCR [69, 75, 79, 82, 83, 85].

On the other hand, reductions in viral infectivity can be investigated by performing virus rescue in cell culture to determine PFUs or TCID_50_ from samples taken before and after the mouthwash to be tested [25, 69, 75, 79, 85]. However, this method is rather complicated and time as well as cost intensive [25]. Furthermore, there is a high probability of negative culture results even in baseline samples because successful virus rescue is only expected from high viral loads above 10^6^ viral RNA copies per mL [86–88]. Accordingly, there are only very few RCTs investigating reductions in viral infectivity of SARS-CoV-2 following use of a preprocedural mouthwash, as summarized in Table 1.

**Table 1:** RCTs investigating reductions of viral infectivity of SARS-CoV-2 after use of a preprocedural mouthwash

Study	Groups	No. of patients (No. of patients with successful assessment of viral infectivity)	Sampling time points	Results Viral load (RT-qPCR)	Assessment of viral infectivity	Results Viral infectivity
Barrueco et al. [85]	- 2% PVP-I - 1% H_2_O_2_ - 0.07% CPC - 0.12% CHX - Placebo	54 (29)	- Baseline - 30 min - 60 min	- No significant reduction in viral load for PVP-I, H_2_O_2_, CPC, or CHX at 30 or 60 min - Significant decrease of viral load for placebo 60 min after rinsing	Virus rescue in cell culture	Significant decrease of 1.5 log genome copies/mL in CPC group 60 min after gargling
Alemany et al. [83]	- 0.07% CPC - Placebo	105 (80)	- Baseline - 60 min - 180 min	No significant reduction in viral load in both groups	Quantification of SARS-CoV-2 nucleocapsid protein by modified ELISA	Significantly increased level of SARS-CoV-2 nucleocapsid protein 60 and 180 min after gargling in test group
Tarragó-Gil et al. [89]	- 0.07% CPC - Placebo	80 (79)	- Baseline - 20 min	No significant reduction in viral load in both groups	Quantification of SARS-CoV-2 nucleocapsid protein by modified ELISA	Significantly increased level of SARS-CoV-2 nucleocapsid protein 120 min after gargling in test group
Meister et al. [75]	- 0.1% BAC - Placebo	24 (6)	- Baseline - 15 min - 30 min	No significant reduction of viral load for both groups at all time points	Virus rescue in cell culture	Mild non-significant decrease of viral infectivity in test group
Bonn et al. [90]	- 0.05% CHX and 0.05% CPC - Placebo	61 (15)	- Baseline - 30 min	Slight (0.5 log_10_) decrease of viral load in both groups	Virus rescue in cell culture	Significant decrease by 1.4 log_10_ 30 min after gargling in test group

Barrueco et al. assessed the antiviral efficacy to SARS-CoV-2 of four commercially available mouthwashes containing 2% PVP-I, 1% H_2_O_2_, 0.07% CPC, or 0.12% CHX as active ingredients in a placebo-controlled RCT [85]. They obtained saliva specimens at baseline and 30 and 60 min after gargling with the mouthwash. Subsequently, they evaluated the viral infectivity by virus rescue in cell culture. Sixty minutes after the CPC-containing mouthwash, a significant decrease of 1.5 log genome copies/mL was found but no significant reduction for the other antiseptics and no significant differences in all groups after 30 min [85].

These results are in accordance with those from the placebo-controlled RCT by Alemany et al., investigating the efficacy of a commercially available mouthwash containing 0.07% CPC [83]. They obtained saliva specimens at baseline and 1 and 3 h after gargling and instead of analyzing viral infectivity by virus rescue in cell culture, they used the modified ELISA for the SARS-CoV-2 nucleocapsid protein described above. Accordingly, the increased detection of nucleocapsid protein is equal to the destruction of the viral envelope by CPC and therefore a decreased viral infectivity [77]. They observed a significant increased level of SARS-CoV-2 nucleocapsid protein 1 and 3 h after the CPC-containing mouthwash in contrast to the placebo group [83].

Similar results were obtained by Tarragó-Gil et al. in their placebo-controlled RCT in 80 patients investigating the efficacy of the same commercially available mouthwash containing 0.07% CPC as described above [89]. They collected saliva specimens at baseline and 2 h after gargling. Using the modified ELISA for the SARS-CoV-2 nucleocapsid protein, they showed a significantly increased detection of the nucleocapsid protein in the salivary specimens 2 h after rinsing, indicative an increase in decomposed virus and decreased infectivity [89].

In the study by Meister et al., a wide range of antiseptics was first evaluated in vitro, whereupon 0.1% BAC was chosen as active ingredient being applied in a placebo-controlled RCT [75]. Samples were taken before and 15 or 30 min after the test or placebo mouthwash and viral infectivity was evaluated by virus rescue in cell culture. While the reduction of SARS-CoV-2 infectivity in vitro reached up to more than 3 log_10_, the results of the clinical trial only showed a mild non-significant decrease on viral infectivity at either post-rinse period, which may have also been related that virus rescue in cell culture was only successful for a rather low sample size [75].

Bonn et al. conducted a placebo-controlled RCT investigating a commercially available mouthwash containing the combination of 0.05% CPC and 0.05% CHX in SARS-CoV-2 positive patients [90]. Oropharyngeal specimens were obtained at baseline and 30 min after gargling and the viral infectivity was analyzed via by rescue in cell culture and determination of TCID_50_. When comparing viral infectivity at baseline and 30 min after the test mouthwash, there was a significant decrease of TCID_50_ by 1.4 log_10_ PFU/mL after gargling with the test mouthwash containing CPC and CHX as opposed to no significant differences in the placebo group [90].

Based on the data from the above-mentioned RCTs, there is growing evidence that use of preprocedural mouthwashes containing CPC can decrease infectivity in the saliva of SARS-CoV-2 positive individuals [83, 85, 89, 90]. Nevertheless, it is not clear yet to what extent this temporary decrease in viral infectivity found in clinical trials is associated with relevant reductions in the risk of transmission of SARS-CoV-2 [25].

## Potential risks and side effects associated with regular antiseptic use

Despite the positive effects of preprocedural mouthwashes described above, possible risks and side effects must be taken into account. While these are mainly to be considered when antiseptic mouthwashes are prescribed or recommended for longer periods, this applies only marginally to the decision for or against preprocedural mouthwashes, since the latter are used in dental practice only before appointments, but not on a daily basis during regular oral care. Nevertheless, possible risks and side effects of regular antiseptic use will be summarized below.

For instance, it is well known that regular use of mouthwashes containing CHX can lead to staining of teeth and tongue, mucosal irritations, or taste alterations [91, 92], which has also been described for CPC, but to a lesser extent [93]. For PVP-I, concerns about resorption in thyroid glands and potential release in the maternal circulation limit its use in certain patient groups, e.g., those with diseases of thyroid glands or pregnant women [94]. Eventually, the traditional EO formulation contains ethanol as a solvent for the essential oils at a relatively high concentration of 26.9%, whose potential side effects have been discussed critically in the literature [95–97], resulting in the marketing of alcohol-free versions about one decade ago [98].

Furthermore, it must be kept in mind that the frequent use of antiseptics like CHX and CPC may also exert some negative effects in terms of potentially detrimental ecological shifts in the oral microbiota [99]. There is evidence from several in vitro as well as in vivo studies that regular use of antiseptic mouthwashes reduces the diversity in oral biofilms and leads to shifts in microbial composition [100–104]. For instance, when we treated 3-day-old microcosm biofilms inoculated from human saliva twice daily with CHX or CPC for a period of 7 days imitating regular use of a mouthwash, we found ecological shifts toward rather caries-associated saccharolytic taxa such as *Streptococcus* spp., *Neisseria* spp., *Schaalia* spp., and *Granulicatella* spp. in the CHX group, while there was enrichment of rather gingivitis-associated taxa like *Fusobacterium* spp., *Leptotrichia* spp., and *Selemonas* spp. in the CPC group [100]. Likewise, Chatzigiannidou et al. reported an ecological shift resulting in a microbial community dominated by streptococci and increased lactate production, when they treated biofilms formed in vitro from 14 species with CHX for 5 min per day over a period of 3 days [101]. In a clinical trial investigating the effects of a CHX-containing mouthwash on the composition of the salivary microbiota in 36 healthy individuals over a period of 7 days, Bescos et al. observed an increase in the relative abundance of caries-associated taxa such as *Streptococcus* spp., *Neisseria* spp., and *Granulicatella* spp. [105].

Besides these potential changes in microbial oral ecology, regular exposure of bacteria to subinhibitory concentrations of antiseptics may further lead to phenotypic adaptation mediated by transcriptomic regulations or even to development of genetically determined resistance toward these antiseptics, potentially associated with cross-resistances to antibiotics [27, 32, 106–109]. Accordingly, several studies have shown that bacteria can adapt upon multiple exposure to subinhibitory concentrations of CHX and CPC [106–108]. We could recently show by RNA-Seq. that treatment of *Streptococcus mutans* with a subinhibitory concentration of CHX led to significant changes in gene expression and regulation of pathways, mainly associated with oxidative stress, biofilm formation, and efflux pumps [109]. Nevertheless, the underlying mechanisms of antiseptic adaptation or resistance are still quite unclear and need further research to unveil the actual clinical relevance [27, 32].

## Conclusion

In summary, clinical data are consolidating that preprocedural mouthwashes containing antiseptic agents can help to temporarily reduce the bacterial or viral burden in the oral cavity or in dental aerosols. Therefore, their use can be recommended as part of a bundle of measures for protection of dental HCPs despite some ambiguities remaining and in view of potential risks and side effects. When choosing an antiseptic agent, it should be considered that the largest available data basis exists for CHX, CPC, EO, or combinations thereof. These recommendations are also in line with those provided by a German S1 guideline from 2021 on how to deal with dental patients when exposed to aerosol-borne pathogens [59].
